# Deep learning assisted mechanotyping of individual cells through repeated deformations and relaxations in undulating channels

**DOI:** 10.1063/5.0077432

**Published:** 2022-02-16

**Authors:** Cody Combs, Daniel D. Seith, Matthew J. Bovyn, Steven P. Gross, Xiaohui Xie, Zuzanna S. Siwy

**Affiliations:** 1 Department of Physics and Astronomy, University of California Irvine, Irvine, California 92697, USA; 2 Department of Chemistry, University of California Irvine, Irvine, California 92697, USA; 3 Developmental and Cell Biology, University of California Irvine, Irvine, California 92697, USA; 4 Department of Computer Science, University of California Irvine, Irvine, California 92697, USA; 5 Department of Biomedical Engineering, University of California Irvine, Irvine, California 92697, USA

## Abstract

Mechanical properties of cells are important features that are tightly regulated and are dictated by various pathologies. Deformability cytometry allows for the characterization of the mechanical properties at a rate of hundreds of cells per second, opening the way to differentiating cells via mechanotyping. A remaining challenge for detecting and classifying rare sub-populations is the creation of a combined experimental and analysis protocol that approaches the maximum potential classification accuracy for single cells. In order to find this maximum accuracy, we designed a microfluidic channel that subjects each cell to repeated deformations and relaxations and provides a comprehensive set of mechanotyping parameters. We track the shape dynamics of individual cells with high time resolution and apply sequence-based deep learning models for feature extraction. In order to create a dataset based solely on differing mechanical properties, a model system was created with treated and untreated HL60 cells. Treated cells were exposed to chemical agents that perturb either the actin or microtubule networks. Multiple recurrent and convolutional neural network architectures were trained using time sequences of cell shapes and were found to achieve high classification accuracy based on cytoskeletal properties alone. The best model classified two of the sub-populations of HL60 cells with an accuracy over 90%, significantly higher than the 75% we achieved with traditional methods. This increase in accuracy corresponds to a fivefold increase in potential enrichment of a sample for a target population. This work establishes the application of sequence-based deep learning models to dynamic deformability cytometry.

## I. INTRODUCTION

Mechanical properties of cells such as the ability to deform when an external force is applied are directly linked to the structure of a cell’s cytoskeleton. Changes in cytoskeleton have been correlated with cellular differentiation,^1^ malignant transformation,^2^ formation of biofilms,^3^ and even COVID-19 pathology.^4^ Consequently, probing mechanical properties offers a label-free method to learn about a cell’s state such as its homeostasis or pathological conditions.

A multitude of techniques have been reported to probe the mechanical properties of cells. Conventional methods include atomic force microscopy,^5,6^ optical tweezers,^7^ and micropipette aspiration.^8^ While these approaches are able to accurately measure mechanical properties of single cells, their throughput of roughly 1–10 cell(s) per minute is significantly slower than the ∼10 000 cells per second achieved by most flow cytometers, which is needed to determine heterogeneities in cell populations and for single cell classification.

In recent years, various microfluidic-based methods have been shown to measure the deformability of cells with significantly improved throughput, closing the gap between flow cytometry and mechanical phenotyping methods. In one class of methods, cells are squeezed through constrictions smaller than the cell’s diameter.^9–12^ The passage time of such strongly deformed cells has been shown to depend on the cells’ mechanical properties, with a faster passage corresponding to more deformable cells. This approach was applied to different cell lines, including cancer cells, stem cells, and red blood cells.^11,13–21^ The transit through the channel is measured by recording electrical impedance or optical signals that enable one to relate parameters like transit time to the deformability and, in certain cases, measure quantities like Young’s modulus.^22^ However, often with these methods, it is difficult to disentangle deformation and surface friction.

Another class of microfluidic approaches utilizes channels that are wider than the cells to be analyzed, where the passing cells are subjected to hydrodynamic forces, and deformations of individual cells are probed optically. Two notable approaches include extensional deformability cytometry (xDC)^23^ and real-time deformability cytometry (RT-DC).^24^ In xDC, a cross-channel is used to deform cells at ultrafast flow rates reaching ∼1000 μl min ${}^{-1}$. xDC has been used to classify malignant pleural effusions, differentiate multiple stem cells, and identify transitions in the cell cycle. The authors showed that deformation kinetics, among other features, are important in the classification of induced pluripotent stem cells (iPSCs).^25,26^ Utilizing their full set of rheological and morphological features, Masaeli *et al.* demonstrated the ability to classify between iPSCs and differentiated iPSCs with accuracies up to 95% using support vector machines (SVMs).^26^ RT-DC, which is based on a straight, narrow microfluidic channel, operates at significantly lower flow rates than xDC ( ∼1 μl min ${}^{-1}$) and induces constant shear stresses allowing cells to reach a steady state deformation. RT-DC quantifies deformability using steady-state images of the deformed cells captured at the end of the microfluidic constriction and is linked to a physical model to calculate Young’s modulus.^27^ RT-DC has also been recently extended to probe the cells’ deformation kinetics as they approach steady state.^28^ RT-DC has been shown to classify reticulocytes from mature red blood cells with an unsupervised approach with a mean accuracy of ∼74%.^29^

While these approaches have shown great promise and success for a variety of problems, the classification of the cell populations relies heavily on morphological and size-based features. While these features are useful in practical applications, the discriminative power of deformability cytometry on cell populations that differ in mechanical properties alone has not been fully explored. Additionally, the use of deep learning models, particularly convolutional and sequence-based models, provides higher classification potential than traditional machine learning models.^30,31^ In this paper, we propose to enhance classification accuracy by maximizing mechanotyping information through subjecting individual cells to repeated deformations and relaxations by hydrodynamic forces as well as through the application of deep learning methods. As the videos are recorded with the time resolution of at least 11 000 frames per second, we can probe the deformation dynamics with high precision. The dynamic observation reveals quantitative insights into the deformation/relaxation processes and provides a comprehensive mechanical fingerprint of each cell. The channel contains a cavity flanked by two narrower regions,^32^ with widths that at any position are wider than the cells to be analyzed. Our technique operates at a throughput comparable to the throughput of RT-DC that enables observations of hundreds of cells per minute and yet probes the cells sufficiently slowly to observe cytoskeletal changes.^33^ Inducing repeated deformation and relaxation by hydrodynamic forces is a natural progression on the way to increase the fidelity of cells characterization and classification based solely on mechanotyping.^34,35^

HL60 cells before and after treatment with either Cytochalasin D (cytoD) or Nocodazole (Noco) were used as a model system to probe the classification potential of our method. Both chemicals were found to perturb HL60 deformability such that cytoD-treated HL60 cells (HL60d) were more and Noco-treated HL60 cells (HL60n) were less deformable than untreated HL60. Most importantly, cells belonging to the three sub-populations, HL60, HL60d, and HL60n, have the same average size, thus enabling us to test our classification strategy based on mechanical properties alone. Using these sub-populations subjected our method to a very challenging test, since classification is often aided by cells’ size.^25,36^

The analysis of the recordings was supported by machine learning approaches with gradually increased levels of expressive power. We show that the mechanotyping dynamic features our method provides enable a significant increase in the classification accuracy of the HL60 populations when compared to using any single feature alone. We also calculated Shapley values, a technique known from the economic game theory, to probe which deformability parameters contributed most to the classification accuracy.^37,38^ A significant improvement in classification accuracy was further observed when the time series of deformations was used as an input into deep learning models such as recurrent neural networks (RNNs). Our deep learning methods utilizing time-based sequences of features showed an increase in classification accuracy to 90%, from the 75% accuracy we observed with the random forest (RF). This increase in accuracy translates to an order of magnitude increase in the potential ability to enrich a sample for a rare population of cells (supplementary material, Note 1). Most importantly, a convolutional neural network (CNN) was used in conjunction with an RNN to utilize sequences of binary masks as input features. We also discuss the trade-off between interpretability achieved with more traditional machine learning approaches and accuracy that is offered by deep learning. Not only has such a comparison not been discussed before, deep learning has not been applied to deformability cytometry for classification based solely on mechanical properties.

## II. METHODS

### A. Cell culture

HL60 (ATCC CCL-240) cells were cultured in suspension with Iscove’s Modified Dulbecco’s Medium (IMDM) supplemented with 10%(v/v) FBS (Fisher brand) and 1% (v/v) penicillin-streptomycin and maintained in an incubator at $37{\;}^{{}^{\circ}}$C with 5% ${\mathrm{CO}}_{2}$. Cells were passaged through dilution every 2–3 days to maintain a density between 10 ${}^{5}$ and 10 ${}^{6}$ cells ml ${}^{-1}$. Prior to experiments, cells were centrifuged at 112 relative centrifugal force (RCF) for 5 min and resuspended to concentrations of 2–3 $\times 10^{6}$ cells ml ${}^{-1}$ in PBS with 1% (w/v) methylcellulose (Spectrum 4000CP).

To create sub-populations of HL60 cells with perturbed actin and microtubule networks, cells were incubated for 10 min in 1 μM cytochalasin D (cytoD) (Sigma) or for 1 h in 10 μM nocodazole (Noco) which had been diluted 10x from stock solution with dimethyl sulfoxide (DMSO).^39^ Cells were spun down at 112 RCF for 5 min and resuspended in 1% (w/v) methylcellulose solution.

### B. Channel preparation and data acquisition

Microfluidic channels were prepared with a master mold using standard photolithography with negative SU-8 photoresist (Kayaku Advanced Materials Inc.). The channel used in the experiments was 150 μm long, with three equal length sub-regions (50 μm) with widths of 25, 50, and 25 μm. The height of the device was 20 μm along the whole length. The full chip geometry can be found in the supplementary material (Note 2). 184-Sylgard polydimethyl siloxane (PDMS) was pipetted over the SU-8 master and baked for ∼4h at 75 ${}^{{}^{\circ}}$C. Channel inlets and outlets were created with a 1.5 mm biopsy punch. PDMS channels were cleaned and dried with isopropyl alcohol, methanol, and water before being bonded to a glass coverslip using a corona discharge wand (ETP). Methanol was used last before drying due to its low boiling point to ensure no residual alcohol remained in the device. Bonded PDMS/glass samples were heated for an additional hour at 90 ${}^{{}^{\circ}}$C to promote further adhesion.

Cells were suspended in methylcellulose solution to prevent settling to the bottom of the container. The solution was then pumped through a microfluidic channel using a Genie-plus syringe pump (Kent Scientific) at a rate of 1 μl min ${}^{-1}$. Cells were focused laterally in the channel using a sheath flow geometry with a flow rate of 2 μl  min ${}^{-1}$. The sheath and core flows were allowed to equilibrate for 10 min before data were taken. Cells were illuminated using a high-powered Red Amber (613 nm) 36 W LED array (PT-121-RAX Luminus, Inc.) and imaged at 10× magnification in brightfield on an inverted microscope. A Chronos 1.4 high-speed camera (Krontech) imaged passing cells at a frame rate of ∼11000 fps with 1 ms exposure time and a resolution of 880×140 to encapsulate the full channel length. The size of each pixel is 0.26μm/pixel. Maximum blurring induced by cell movement is ∼0.1 μm. Eight second videos were recorded and saved which require ∼15 min to offload from camera memory. In order to ensure model generalizability, in total, ∼3500 cell trajectories were recorded and analyzed. The data presented were collected over 10 technical replicates of HL60 (4 biological replicates), 8 technical replicates of HL60d (4 biological replicates), and 6 technical replicas of HL60n (2 biological replicates).

### C. Detection, segmentation, and tracking

A convolutional neural network (CNN) was trained to identify frames containing cells to reduce the computational time of processing the large number of frames generated by the high-speed camera. Frames labeled as containing cells were then passed to a segmentation MASK-RCNN network that was used to segment cells from images and fit masks. MASK-RCNN can learn to accurately segment cells with differing focusing conditions and is able to segment multiple cells in a single frame. The Matterport implementation^40^ was used with Tensorflow 2.2 due to its wide code availability and its support for Tensorflow. The network was trained using a NVIDIA 1070TI on a hand labeled dataset of ∼300 images of HL60 cells across multiple independent experiments using VGG Image annotator 1.0. The network was initialized with weights from the COCO dataset and the architecture was modified in order to increase the output resolution of the predicted masks. The training schedule consisted of training head layers for 20 epochs at a learning rate (LR) of 10 ${}^{-3}$, 50 epochs training 4+ layers at LR/10, and 50 epochs training all layers at LR/10. Training curves and further details can be found in the supplementary material (Note 3). A custom tracking algorithm was written in python to follow individual cell trajectories.

### D. Calculation of features

Detected events with impossible trajectories (i.e., no event will begin in the middle of the channel) were discarded. To ensure the fits are accurate, a convex hull was fit to the cell mask and events were filtered out where a single frame had a ratio >1.1 between the original and convex hull fit. Detected particles with radii equal to three standard deviations from the mean were not included in the analysis, as they often contained cell/channel debris or clumps of multiple cells. Ellipses were fit to the detected masks and the aspect ratio of the ellipse was used to describe the cell deformation, AR. A full description of deformation parameters can be found in the supplementary material (Note 4).

### E. Comsol simulation

A finite element simulation was conducted using Comsol Multiphysics 5.3 to simulate the velocities and stresses experienced in the undulating channel. A simplified 3D model of the channel was modeled in the laminar flow module using the Navier–Stokes equation with creep flow in the steady state. An extra fine meshing was chosen for the area near the narrow section of the channel, and normal meshing was chosen for the reservoir.

### F. Machine learning model training

Of all the cell trajectories recorded, 3552 were suitable for classification. They were distributed as 1114 trajectories of HL60, 1122 trajectories of HL60d, and 1316 trajectories of HL60n.

The RF model and SVM were created and trained using python 3.6 and scikit-learn. The data were standardized, shuffled, and split according to the following ratio: 70:15:15 for train, validation, and test, respectively.

The GRU and the CNN-GRU models were created and trained in python 3.6 using Keras and Tensorflow 2.2 libraries. The hyperparameters were optimized over 175 epochs using the test set. Model performance was assessed using a hold-out test set, in addition to fivefold cross-validation. Additional details on both model architectures can be found in the supplementary material (Notes 5 and 6).

### G. Data preprocessing for sequential models

Sequences of aspect ratio, perimeter, deformability, and area were selected as inputs for the GRU model. Data previously filtered were first aligned so that all sequences started and ended at a true x-position of −30 and 170μm, respectively. Data were then padded to length 50 and were shuffled and split according to the following ratio: 70:15:15 for train, validation, and test, respectively.

For the sequential models using image data, CNN-GRU, masks were first cropped to 96×96. An ellipse fitted to the mask shape was created using scikit-image and added to the second image channel. Each sequence of these two-channel images was padded to length 50. The data were shuffled and split according to the following ratio: 70:15:15 for train, validation, and test, respectively.

### H. Code availability

The python scripts implemented for data processing and training machine learning models are available at https://github.com/siwylab/time-series-dc.

## III. RESULTS AND DISCUSSION

### A. Channel design and data acquisition

**FIG. 1.**
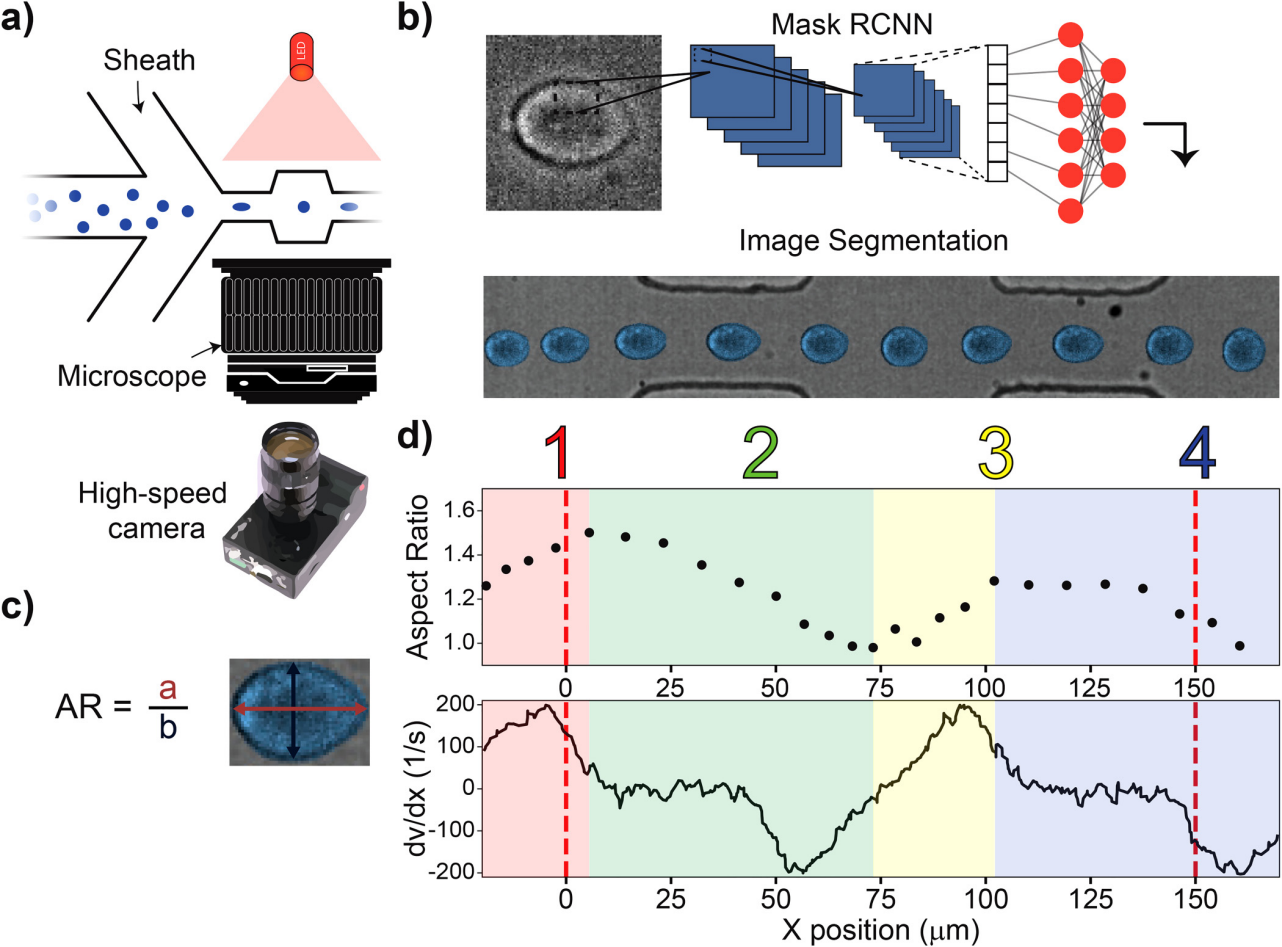
Principles of repeated mechanotyping. (a) Channel design utilizing sheath flow, a high-powered LED, and a microscope. The microfluidic channel that enables characterization and classification contains a cavity placed between two narrow zones. (b) Data are captured by a high-speed camera, creating videos at 11 k fps. Cell borders are detected and fit using Mask-RCNN. (c) The cell deformation, AR, was quantified as the ratio of two axes of an ellipse that approximates the cell’s shape. (d) (Top) The aspect ratio vs position relative to channel entrance of a single cell as it passes through the channel. (Bottom) COMSOL simulation showing the derivative of velocity vs channel position, which is proportional to the shear stress. Region 1 (R1) and Region 3 (R3), denoted by red and yellow regions, are where the cells undergo deformation. Region 2 (R2) and Region 4 (R4), denoted by green and blue regions, are where the cells undergo relaxation.

We designed a microfluidic channel that subjects individual cells to repeated compressions and relaxations. The multiple deformations of the cells are induced by the channel shape, specifically the presence of a cavity and two narrow zones. The varying channel width is expected to create inhomogeneous velocity profiles leading to temporal changes in the cells’ shape.^41^ To test this channel design for mechanotyping, we pumped a suspension of HL60 cells (ATCC-240) in methylcellulose (1% w/v) solution through a channel of consecutive constrictions of length equal to 50 μm (a total length of 150 μm) and widths of 25, 50, and 25 μm at a flow rate of 1 μl min ${}^{-1}$. The more viscous methylcellulose solution induces larger deformations than buffer solution and prevents sedimentation of cells. Sheath flow focusing was used to ensure that all cells passed through the channel along its center axis and experienced the same forces. The microfluidic chip was placed on a 10 × magnification inverted microscope. To enable probing dynamics of cells’ shape at this high flow rate, we customized the microscope to allow for high-speed imaging by replacing the light source with a high-powered LED array and accompanying 3D printed mount [Fig. 1(a)]. A high-speed camera was adapted to fit the microscope, and videos of translocating cells were recorded at 11 000 frames per second, resulting in ∼30 data points over 150 μm channel and revealing the shape dynamics as a cell moves along the channel axis.

After videos had been collected, the data were processed with a number of python scripts (supplementary material, Note 7). The first script encompasses a lightweight CNN that filters out empty frames and reduces the amount of data to be processed by ∼80%. Next, we employed a modified architecture of the Matterport^40^ implementation of Mask-RCNN,^42^ which is used to detect cell regions and their associated mask. Our version of Mask-RCNN is trained on 300 hand-labeled images of cells with varying levels of focus (supplementary material, Note 3). The masks detected by this network are then processed using a custom tracking algorithm to find trajectories of individual cells. Figure 1(b) shows subsequent snapshots of one cell as it passes through the channel. MASK-RCNN enables us to track multiple cells in the same frame, allowing a high density of cells ( $5\times 10^{6}$ cells ml ${}^{-1}$) to be used in the experiments. We found that the undulating channel design results in complex dynamics of the cell’s shape and leads to regions of differing deformations. Specifically, the cell underwent a strong deformation at the entrance of the channel and in the first narrow constriction; the cell then relaxed to a spherical shape in the cavity and started to deform again when approaching the second narrow constriction. As seen in Fig. 1(d), we define the regions where the cell undergoes deformation as region 1 (R1) and region 3 (R3). The regions where the cell undergoes relaxation are denoted as region 2 (R2) and region 4 (R4).

We find that the shape of the deformed cells in our device can best be described as an ellipse where the deformation (D) is defined as the aspect ratio (AR) of the two axes of the ellipse: the axis parallel to the channel axis and the perpendicular axis [Fig. 1(c)]. The relative deformability (rD) is the difference between D at a given point in the trajectory and D in the cavity, where the cells undergo no deformation. rD=0 corresponds to lack of deformation relative to unperturbed cell shape, whereas rD>0 corresponds to an extension along the channel axis. Figure 1(d) summarizes how the magnitude of AR evolves as the cell shown in Fig. 1(b) passes through the channel. In order to qualitatively understand the deformation trace, we performed a computational fluid dynamics simulation with COMSOL multiphysics in a cell-free undulating channel using the Navier–Stokes equations with creep flow at experimental flow rates. Shear stress in different parts of the channel can be analyzed through the derivative of velocity in the center of the channel with respect to the axial position shown in Fig. 1(d). The cells experience a large velocity gradient at the entrance of the channel, leading to large stresses and deformations.

**FIG. 2.**
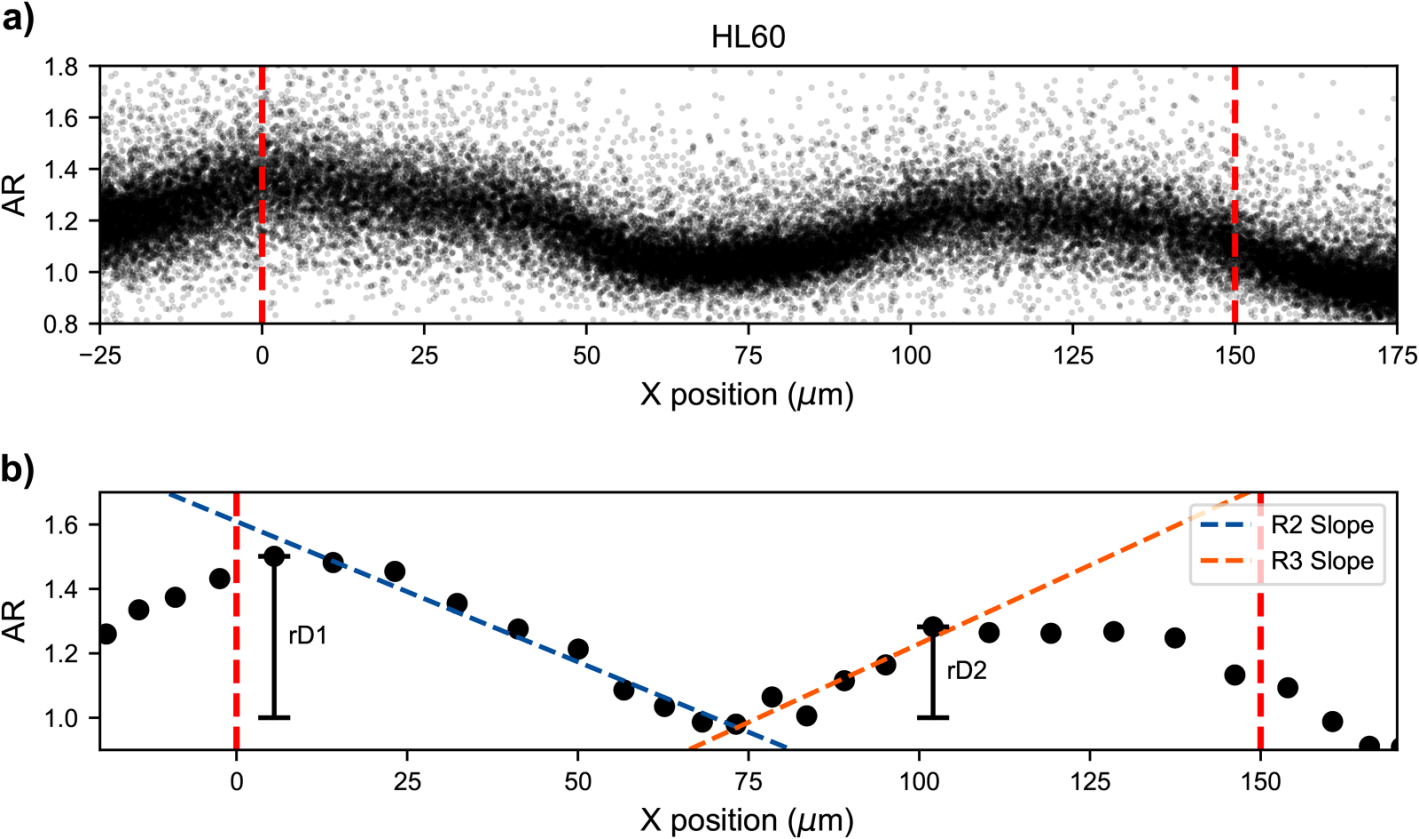
Single cell deformation traces. Deformation dynamics are shown for single cells translocating through the channel. The aspect ratio is determined by the best fit ellipse to the cell mask. Deformation is calculated by the difference between the aspect ratio at a given point and the minimum aspect ratio in the cavity. The x-position along the channel axis is determined by the centroid of the mask. Cells experience a smaller maximum deformation in the second narrow region, as compared to the first narrow zone. Channel inlets and outlets are marked by a red dotted line. Deformation and relaxation occur twice within the channel. (a) Full population of single cell traces of aspect ratio vs position for untreated HL60 cells. (b) Single cell example of parameters that are determined: relative maximum deformations (rD1 and rD2) in the two narrow zones as well as relaxation and deformation slopes (R2 slope and R3 slope).

For the single cell trace shown in Fig. 1(d), we observe a peak deformation of AR=1.49 in the first region (R1), marked in red. The velocity gradient then reaches a steady state value within the first narrow constriction (10–50 μm) and the cell deformation decreases. Before the cell can reach a steady state deformed shape, it enters the cavity where the velocity gradient begins to decrease and then again rapidly increases. The cell returns to a spherical shape, AR=1, where dv/dx=0, which occurs at ∼75μm. The cell then begins to deform again due to the velocity gradient at the entrance of the second narrow constriction, reaching a second peak deformation of AR=1.30 in region three (R3). The maximum deformation observed in the second constriction is lower than the maximum deformation measured in the first constriction. We believe this is because the cell is subjected to positive shear stresses over a relatively short time and distance when transitioning from the cavity to the second narrow region, as compared to the transition from the bulk channel to the first narrow constriction entrance. The distance from the middle cavity, where the shear stress is zero, to the position of peak deformation (and maximum positive shear stress) in the second narrow region is only 25 μm, whereas at the initial inlet the cell begins deforming from positive shear stresses ∼50 μm away from the entrance. Figure 2(a) shows the values of AR for ∼1200 HL60 cells examined in the same conditions. The same trend for all cells has been observed: the cells reached the maximum deformations in the first narrow zone, relaxed to a sphere in the cavity, and underwent another deformation in the second constriction.

Taking advantage of the time series of cells’ positions and shapes, we also quantified the dynamics of deformation and relaxation. To this end, we used a linear model to fit the trace from the peak deformation in the first narrow zone to the position where the cell relaxes to a spherical shape in the cavity (R2), resulting in a slope of $-8.71\times 10^{-3}\;\mu m^{-1}$. The slope describes relaxation dynamics, and we refer to it as R2 slope. A similar analysis can be performed for the cell entering the second narrow zone by fitting a line between the spherical shape in the cavity to the maximum deformation in the second narrow zone, obtaining a value of $9.75\times 10^{-3}\;\mu m^{-1}$. This slope, called R3 slope, describes the deformation dynamics. Figure 2(b) shows example fits of rD, R2, and R3 slopes to a trace of AR evolution for a single cell passing through a channel.

### B. Application of the undulating channel to probe perturbation of actin and microtubule networks

In order to evaluate the sensitivity of our method to detect cytoskeletal perturbations, we continued the experiments with HL60 cells and treated them with cytochalasin D (cytoD) and Nocodazole (Noco). These two chemicals have been previously used to create model populations of HL60 that allowed researchers to evaluate the performance of different mechanotyping techniques.^23,24^ CytoD disrupts actin polymerization^43^ and has been previously shown to increase deformability of HL60.^22,24,39^ Noco targets microtubules causing rapid filament decomposition. For HL60 cells, Noco has previously been found to decrease the ability to deform.^39^ Perturbing different components of the cytoskeleton allowed us to test whether the deformation/relaxation processes from our channels and the chosen flow rate are able to measure changes in whole cell deformation after actin and microtubules disturbance.^33^ The three populations of HL60 cells, untreated, CytoD-treated, and Noco-treated, were suspended in methylcellulose solution and separately passed through our microfluidic channel. After post-processing, passages of more than 1000 cells of each sub-population were captured. Recordings were taken over 10 technical replicates of HL60 (4 biological replicates), 8 technical replicates of HL60d (4 biological replicates), and 6 technical replicas of HL60n (2 biological replicates). The repeated experiments and the amount of cells measured show consistent trends and enable statistical analysis of the various measured parameters across the populations.

**FIG. 3.**
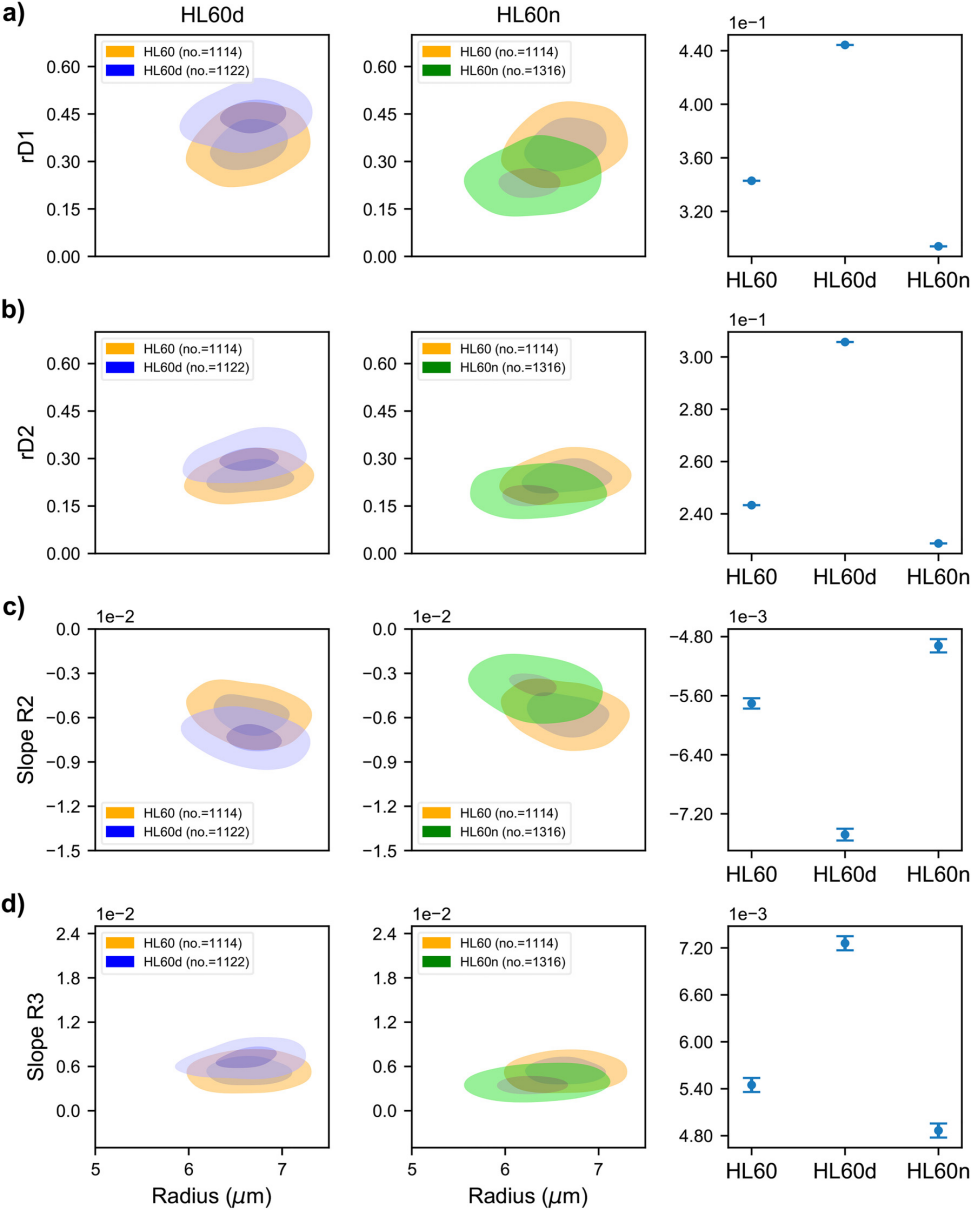
Comparison of measured deformability features between untreated and treated HL60 cells. (a) Contour plot of maximum rD in the first narrow zone (R1) for untreated, cytoD-treated, and HL60n cells. The outer contour represents 50% density and the center contour represents 90% density. The mean of each population is reported where the reported error is the standard error of the mean. (b) Contour plots of maximum rD in the second narrow region (R3). (c) Linear fit slope from maximum deformation in the first narrow region (R1) to relaxation to minimum deformation in cavity (R2). (d) Linear fit slope from relaxed state in cavity (R2) until maximum deformation in the second narrow region (R3).

Figures 3(a) and 3(b) show the rD in the two narrow zones and the two slopes for the three populations [Figs. 3(c) and 3(d)] of HL60 cells. The magnitudes of rD are consistently the highest for the cytoD-treated population, which confirms that the method is sensitive to actin network perturbations. The Noco-treated populations exhibit the lowest mean rD, albeit with a larger variability than the other two populations. These findings are in agreement with earlier reports that used the same cell line and also observed increased (decreased) deformability of the cytoD (Noco)-treated HL60 populations.^39^

Interestingly, the R2 and R3 slopes that represent relaxation and deformation dynamics, respectively, are also the greatest for the cytoD-treated cells, suggesting that these cells are most responsive to external forces. In contrast, the slopes for the less deformable, Noco-treated populations are characterized by significantly smaller magnitudes of both slopes than cytoD-treated and untreated HL60 cells. Though the HL60n had a slower response to the shear stress, they relaxed to a spherical shape in the cavity and deformed again in the second narrow region. It is important to note that the mean diameter of the three populations is nearly identical (supplementary material, Note 8), confirming the cells experienced similar forces.

While the mean values of deformation are significantly different (Fig. 3, third column), there is a significant overlap between the control HL60 and treated cell distributions (Fig. 3, contour plots). This overlap makes it difficult to place an individual cell in one population or the other. We find that no single feature alone can provide accuracy significantly higher than 70% for single cell classification (supplementary material, Note 4). For example, rD1 alone is able to classify between HL60 and HL60d populations at 72% accuracy using a logistic regression model, while the same parameter classifies HL60n with only 57% accuracy. The radius alone can be used to classify up to 56% and 59% for HL60d and HL60n, respectively, as expected from the nearly identical size of the populations. In Secs. III C–III E, we will show that in order to achieve discrimination between the two populations on a single cells basis, multiple features describing the cell deformation (Fig. 3) must be considered simultaneously.

### C. Feature extraction and machine learning model comparison

Our next goal was to utilize the complete mechanotyping fingerprint our method provides, to maximize classification accuracy. As mentioned above, the three populations chosen were nearly identical in size; thus, the differences in mechanical properties provide the only basis for classification. We first investigated the ability of traditional machine learning models to distinguish the two pairs of sub-populations, HL60 vs HL60d and HL60 vs HL60n. To provide a baseline for classification potential, features were first manually extracted from the individual raw time-series data as seen in Fig. 2. A full list of extracted parameters can be found in the supplementary material (Note 4). These derived features were used to train a random forest classification model^44–46^ (RF), which has been previously employed for classifying cytometry data.^47^ The random forest models were implemented using scikit-learn and all relevant hyperparameters were optimized. Other models such as support vector machines (SVMs) were tested and showed similar or less performance compared to RF (supplementary material, Note 9). All models were trained across a mixture of multiple biological and technical replicates, and accuracy results were reported on a held on test set, in order to minimize bias.

**FIG. 4.**
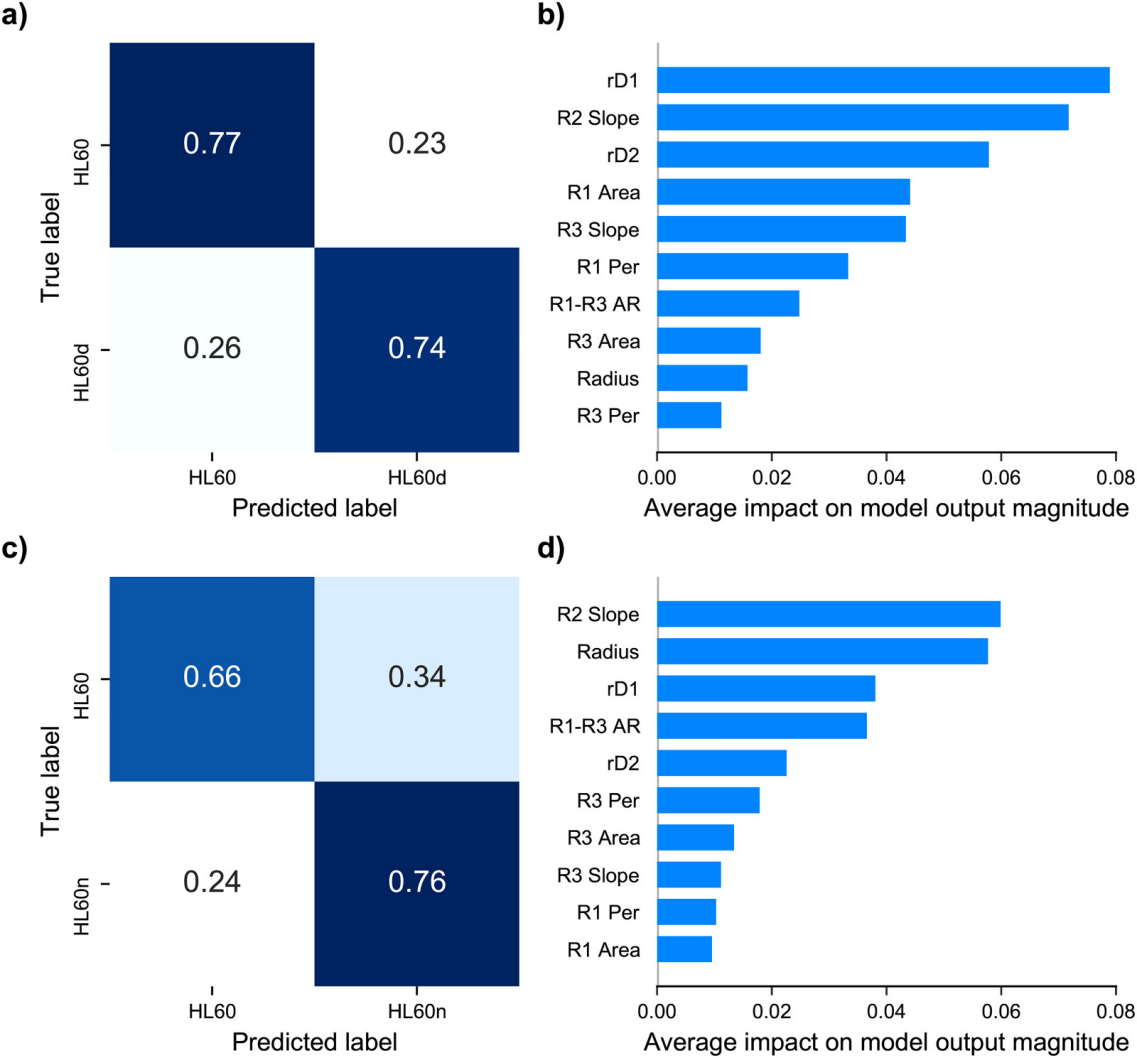
(a) Prediction results of random forest using derived mechanotyping features. (a) Confusion matrix of trained random forest predicting HL60 vs HL60d. The values are normalized by the true label count. Accuracy is equal to the average of diagonal. (b) SHAP feature importance plot obtained using the trained RF model for the HL60 vs HL60d classification. (c) Confusion matrix for random forest trained on HL60 vs HL60n prediction. (d) SHAP feature importance for HL60 vs HL60n.

We found that the trained RF model resulted in 75% classification accuracy for HL60 vs HL60d and 71% classification accuracy for HL60 vs HL60n [Fig. 4(a) and 4(c)]. The lower accuracy of the model trained on HL60n can be attributed to the larger variation in cell radius and deformation of Noco-treated cells. In the next step, we wanted to understand the impact of different mechanotyping features on the RF model’s predictions. To this end, we utilized Shapley values, which were developed for coalitional game theory to inform how to fairly attribute success to the constituent parts.^37^ Thus, Shapley values can help us understand which features are most informative for a single cell classification. In order to make use of this method, the SHAP python library^48^ was employed in conjunction with the trained RF model to attribute overall model performance to individual features [Fig. 4(b)]. The values reported in Figs. 4(b) and 4(d) represent the mean impact for an individual feature in determining cell type. From the Shapley values, we find that the R2 slope and rD1 have the most weight in deciding classification between HL60 vs HL60d. Whereas for the HL60 vs HL60n case, we find the R2 slope to have the highest impact, followed by the radius. The introduction of Shapley values provides interpretability of the mechanotyping data and demonstrates that the region from peak AR to the relaxed state contains the most information for classification. The analysis also revealed the importance of another temporal feature, the R2 slope, in the classification [Fig. 2(b)]. Based on these observations, we hypothesized that classification could be further improved by incorporating the sequential nature of a cell’s deformation into the model design. We then decided to explore deep learning approaches to create a model that can extract shape dynamics.

### D. Deep learning for enhanced classification

**FIG. 5.**
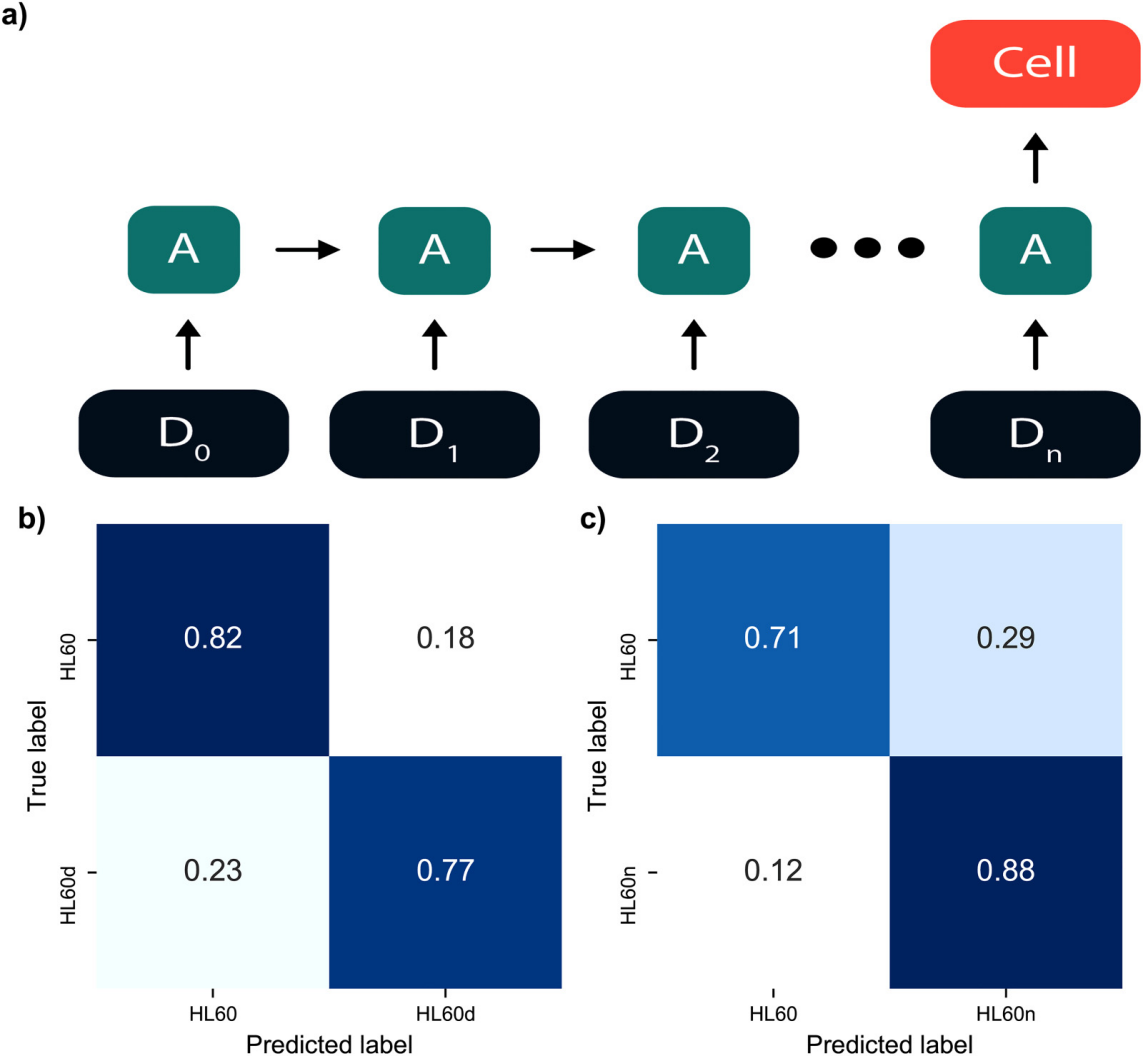
Time-series neural networks applied to mechanotyping features. (a) Outline of recurrent neural network. Time-series deformation data are used as input into GRUs. The output of the network predicts cell phenotype. (b) Confusion matrix for RNN trained on HL60 vs HL60d. (c) Confusion matrix for RNN trained on HL60 vs HL60n.

RNNs are considered the optimal tools for handling sequential data^49^ and are often used for translation and sequential prediction tasks. We chose RNNs since they can approximate a function describing the shape dynamics, which in our case is complex due to the repeated deformations and relaxations caused by the non-linear shear force [Fig. 1(c)]. In short, RNNs function by receiving a single input from the full sequence, processing it, and feeding the output into a copy of itself along with the next time step. We implemented a variant of RNNs called the gated recurrent unit (GRU) and used the sequential time-series features such as aspect ratio, perimeter, and area as inputs to these models. The trained GRU displayed a moderate increase in classification performance, as seen in the confusion matrices in Figs. 5(b) and 5(c), resulting in a 4% accuracy increase for HL60 vs HL60d and an 8% increase for HL60 vs HL60n. Another RNN variant, Long Short-Term Memory (LSTM), was tested but did not perform as well (supplementary material, Note 10). GRU model architecture is presented in the supplementary material (Note 5).

**FIG. 6.**
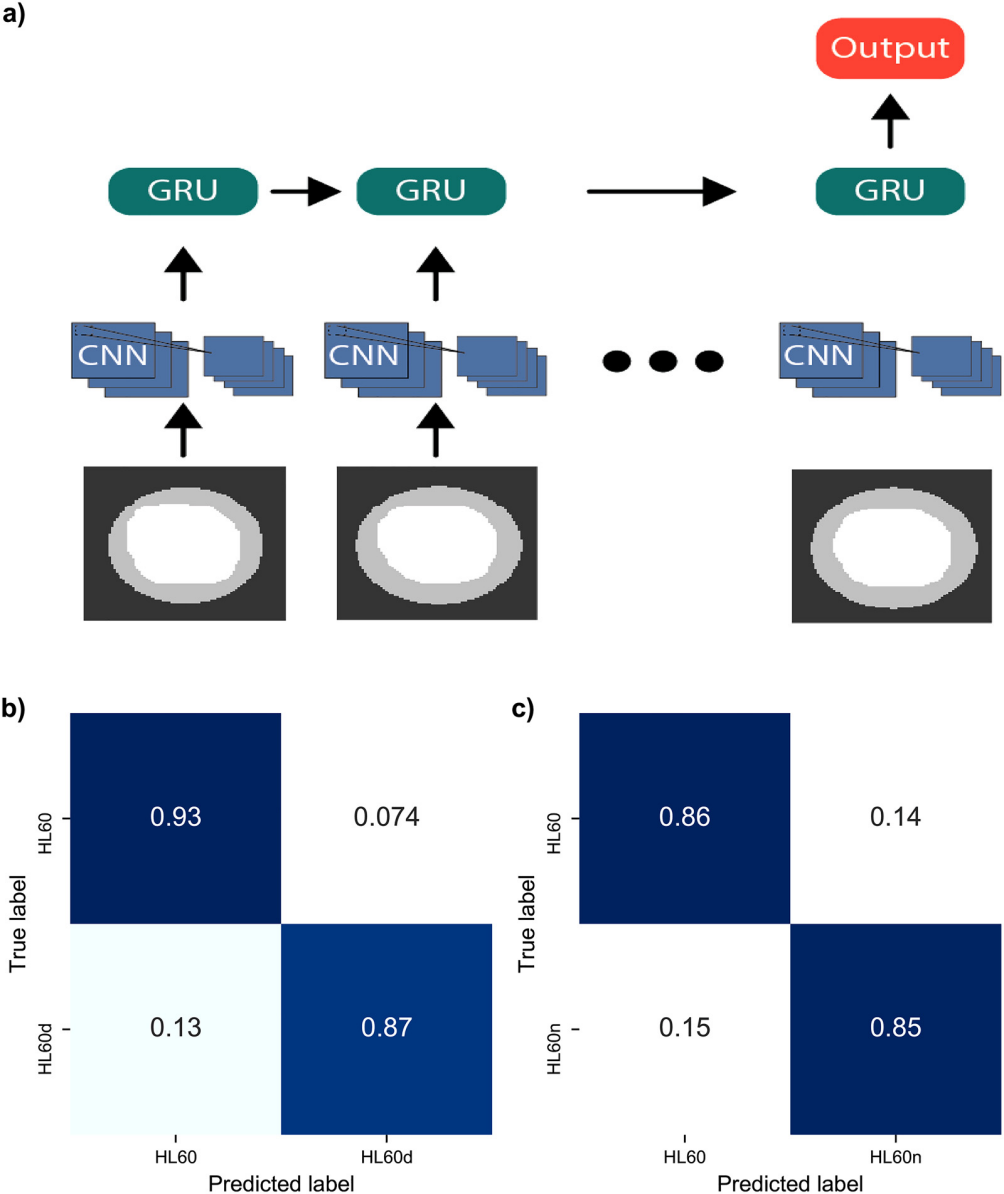
Classification comparison using the sequence of cell masks. (a) General flow of CNN-GRU. Sequences of masks are padded and used as inputs. CNN and GRU layers use identical weights for each time step. (b) Confusion matrix for HL60 vs HL60d. (c) Confusion matrix for HL60 vs HL60n.

Since the application of RNNs yielded improved performance (Fig. 5) compared to the RF models with manually derived features (Fig. 4), we hypothesized that further improvement would require an approach that is not based on hand-selected features (or their combination). We, therefore, sought to improve accuracy by using the binary masks as inputs, obtained using our video-processing algorithm described above (see the sequence of blue shaded regions in Fig. 1). Note that the sequence of the masks is the only input; the derived deformation values from the masks are not used here. To utilize the time series of masks, we have added a number of convolutional layers to the GRU model (CNN-GRU). Convolutional neural networks are known to be very well suited for image-based classification tasks. A schematic diagram of the model is shown in Fig. 6(a).

While CNN-RNNs traditionally use raw frames as inputs, here our input complexity is drastically reduced since segmentation had already been performed. Consequently, the network only learned the shapes of the deformed cells, as opposed to differences in internal morphology or brightness contained in the raw images. Our CNN-GRU architecture was refined by searching different filter sizes for the CNN layers, changing the number of dense and GRU layers, as well as adjusting the dropout rates. The optimized model (supplementary material, Note 6) shows superior performance of the CNN-GRU to both the RF and GRU models [Figs. 6(b) and 6(c)]. Namely, the CNN-GRU enabled an increase in accuracy by 11% to HL60 vs HL60d and an increase of 6% to HL60 vs HL60n, resulting in final classification accuracies of 90% and 85%, respectively. To investigate the potential of over-fitting, fivefold cross-validation was performed in addition to the use of a train, validation, and test split. The fivefold cross-validation resulted in accuracies of 91.4 ±0.6% and 83.7 ±0.9%, with similar results obtained for each training regime. The high validation set performance shows the richness of the deformability dynamics and their potential to aid in classification.

Finally, we wanted to confirm the importance of mechanotyping in the classification of HL60 sub-populations and asked whether these cells could be distinguished only through their morphological features, such as shape, prior to deformations. To this end, a CNN was trained to classify HL60 vs HL60d and HL60 vs HL60n sub-populations using masks from the channel cavity, where no induced deformations are present. The best test accuracy attainable was 65% for HL60 vs HL60d, indicating the poor classification potential of the undeformed shape (supplementary material, Note 11). On the other hand, when the same analysis was performed for HL60 and HL60n, we saw a classification accuracy of 70%, indicating that the Noco treatment affected the cells’ undeformed shape, relative to the control HL60 population. The modified morphology of HL60n could have also contributed to the large increase in accuracy when using the CNN-GRU. Combining mechanotyping and deformation dynamics with the morphology of cells could lead to improved accuracy of cells classification in cases where the initial morphology differs.

### E. Traditional ML with added morphological features vs deep learning

To investigate the gain in performance obtained by deep learning over traditional machine learning methods, we extended the feature set of the manually derived features to include more descriptors of morphology. The added features were calculated in regions R1, R2, and R3 (Fig. 1) and included the central moments of inertia along with Hu moments,^50^ which are a set of seven two-dimensional moments that are invariant to rotations, scaling, and translation (supplementary material, Note 12). Using these new morphological descriptors, along with the previous derived features (supplementary material, Note 4), a new SVM was trained and optimized through a grid search of relevant parameters. The resulting confusion matrix on a held-out test set and SHAP plots were calculated to understand the importance of these added features for classification (supplementary material, Note 12). The classification accuracy between HL60 and HL60d showed a marginal increase in performance (1%) compared to the SVM trained without the additional feature set. This, along with the poor classification of the undeformed shapes (65%) (supplementary material, Note 11), suggests that the HL60 and HL60d cells are very similar morphologically and that deformation-based features are the most informative for classification. The classification of HL60 vs HL60n, on the other hand, was moderately improved by the newly added morphological features ( ∼10%). The corresponding SHAP plot (supplementary material, Note 12d) shows that the Hu moments in R2, where the cells are most relaxed, had the highest average impact on classification. The increase in classification accuracy along with the importance of features in R2 suggests that both morphological and deformation-based features contributed to the classification of HL60 and HL60n.

While the addition of 30 new morphological features demonstrated an increase in SVM classification accuracy, it is impossible to ensure that our set of derived features forms a complete basis to describe all possible shapes the cells assume throughout their deformation and relaxation. Furthermore, we argue that features like the Hu moments, although useful for increasing classification, offer little interpretability and cannot be used to inform future physical models. In cases where one is most concerned with classification accuracy over interpretability, we found that deep learning, in particular, a network with both convolutional and recurrent layers, outperforms traditional methods like an SVM. The classification accuracy of the CNN-GRU was ∼15% higher for HL60 vs HL60d and ∼4% for HL60 vs HL60n. We assert this increase in performance is due to the ability of convolutional layers to generate a more complete feature set, along with the recurrent layers to learn the temporal dependence of the same features. It is important to highlight that the use of deep learning models, like the CNN-GRU described here, currently presents almost no interpretability. However, this comes with a trade-off of improved classification accuracy.

### F. Increased accuracy leads to higher enrichment

To explore the implications of an increased classification accuracy, we considered a hypothetical case in which a researcher has a heterogeneous sample of cells of many mechanical phenotypes and desires to characterize a sub-population within. The sub-population constitutes at most a few percent of the total sample making other, more conventional analysis, such as single cell RNA sequencing and microscopy, difficult. We envision our method can be implemented to identify the population of interest by combining deformability characterization, classification, and in the future sorting. In this case, classification accuracy determines how effectively rare cells can be sorted out of the mixed sample. In the supplementary material (Note 1), we derive a relationship between the classification accuracy and the number of target cells recovered in a sorted sample. We define the enrichment as the increase in the concentration of target cells in the sorted sample. We find that enrichment depends sensitively on classifier performance, increasing exponentially as false positive rate decreases below 0.5, then super-exponentially for false positive rate less than ∼0.2 as shown in Fig. S1 in the supplementary material. This dependence gives context to the importance of the increased classification performance we achieve. Using only morphological features, the classifier performance would allow enrichment of ∼9 times. Using the mechanotyping data but with the inferior random forest classifier would allow an enrichment of ∼12 times. Finally, using the CNN-GRU model would allow an enrichment of ∼58 times (each averaging over prediction performance numbers for HL60d and HL60n, and assuming a rarity of 1/1000). Thus, the CNN-GRU classifier has about five times the potential effectiveness of the random forest classifier for future sorting applications.

## IV. CONCLUSIONS

In summary, we show the application of a channel with an undulating width that induces non-linear forces to individual cells at high throughput. The cells undergo multiple deformations and relaxations that reveal a multitude of information on cellular mechanics. We explore how much information is revealed from this dynamic and non-linear deformation process by comparing the classification accuracies of traditional machine learning models, with derived features, to deep learning models with automatic feature extraction. For the traditional machine learning models, the use of SHAP values unraveled mechanotyping parameters that contributed most to classification, which can be useful in gaining insight into how cell populations differ mechanically. At the cost of interpretability, the deep learning models showed an appreciable increase in classification accuracy, particularly in the case where the two cell populations were very similar morphologically.

While the deep learning characterization and classification are currently performed *post hoc*, similar deep learning models with proper hardware can currently process ∼2000 fps, leading to the possibility for real-time sorting. We envision this work being incorporated into currently existing technologies, such as imaging flow cytometry, and to be especially important for segregating rare cells, including circulating tumor cells^51^ and even cancer stem cells.^52^

Future work will include optimizing channel designs with different widths, lengths, and shapes, as well as extending the deep learning models to include unsupervised classification.

## SUPPLEMENTARY MATERIAL

See the supplementary material for additional information concerning enrichment calculations, feature descriptions, and model architectures and their performance.

## Data Availability

The data that support the findings of this study are available from the corresponding author upon reasonable request.
